# Supplementary material for: Repeated species radiations in the recent evolution of the key marine phytoplankton lineage Gephyrocapsa
Source: Nat Commun. 2019 Sep 17;10:4234. doi: 10.1038/s41467-019-12169-7 (PMC6748936; doi:10.1038/s41467-019-12169-7)
Supplement: Supplementary file 1 — Supplementary Information [file 41467_2019_12169_MOESM1_ESM.pdf]

Supplementary Information

Repeated species radiations in the recent evolution of the key marine phytoplankton lineage Gephyrocapsa

Bendif M, Nevado B, Wong ELY, Hagino K, Probert I, Young JR, Rickaby REM and Filatov DA

Supplementary Figures

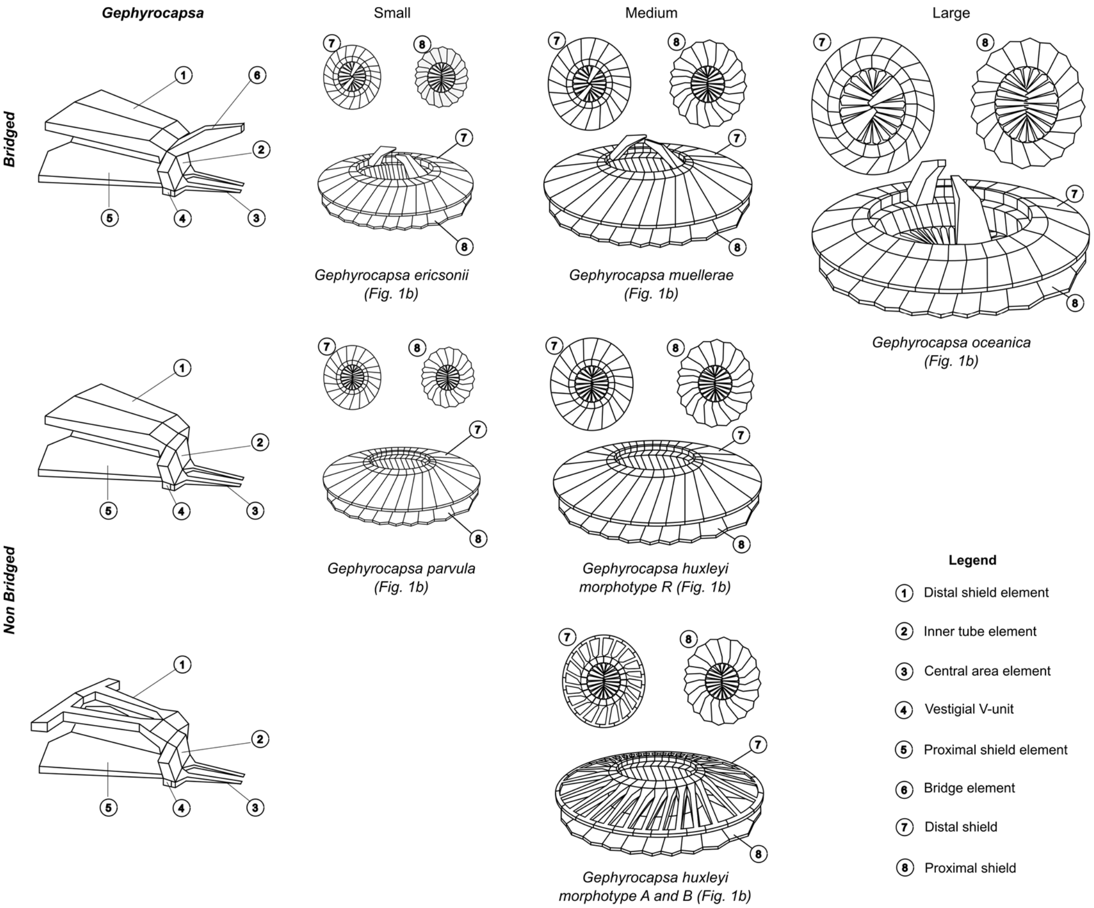

Supplementary Figure 1 | Coccolith structure of representative Noëlaerhabdaceae. (Redrawn from Bendif *et al* 2016[1] by El Mahdi Bendif)**.** Each morphospecies is associated to its respective SEM image in Figure 1b.

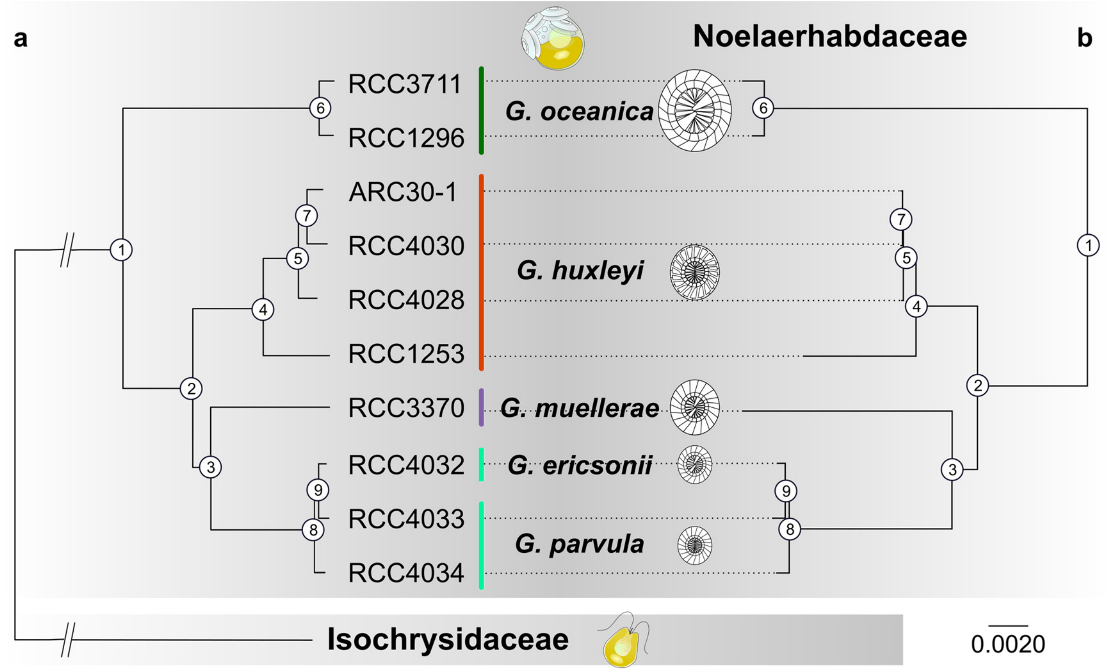

Supplementary Figure 2 | Phylogenetic reconstruction of the species tree of the Noëlaerhabdaceae. (**a**) ML phylogenetic tree of the Noëlaerhabdaceae rooted with both *I. galbana* and *T. lutea* based on an alignment of 1505 concatenated orthologous proteins (948,002 residues, of which 385,081 residues were retained after ambiguous region removal). Both outgroups consistently place the root of the Noëlaerhabdaceae phylogeny on the branch connecting *G. oceanica* with other *Gephyrocapsa* species. (**b**) Species tree of the Noëlaerhabdaceae based on sequenced *Gephyrocapsa* strains, inferred from a concatenated matrix of 2137 contigs, accounting for 4,105,458 phylogenetically informative positions. Both trees are drawn to scale, with branch lengths measured in number of substitutions per site (scale bar). All nodes (numbered from 1 to 9) in both trees have bootstraps values of 100%.

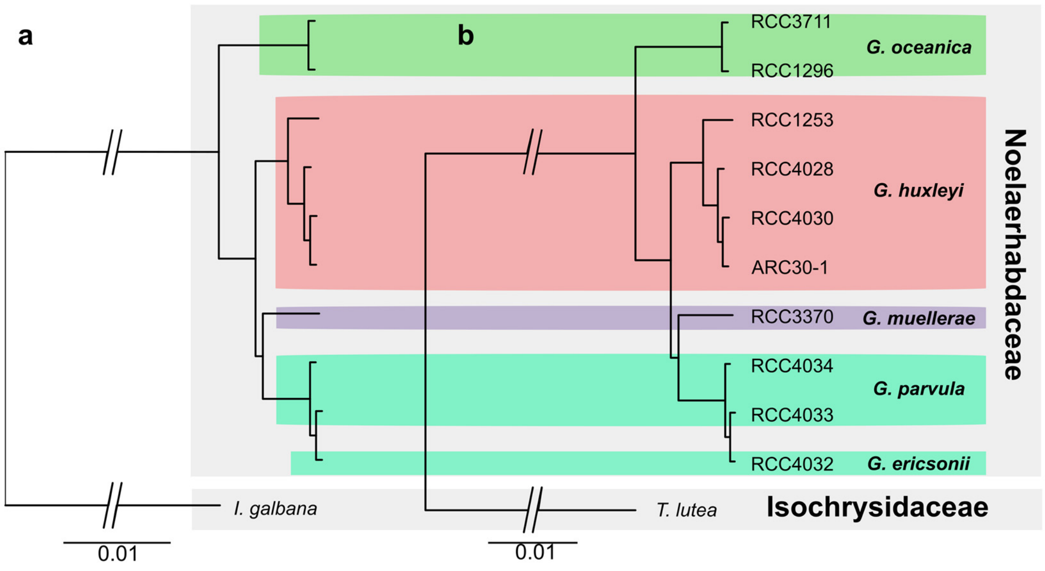

Supplementary Figure 3 | Finding the root for the Noëlaerhabdaceae phylogeny. (**a**) ML phylogenetic tree of the Noëlaerhabdaceae rooted with *I. galbana* based on an alignment of 2870 concatenated orthologous proteins (1,579,905 residues, of which 709,985 residues were retained after ambiguous region removal). (**b**) ML phylogenetic tree of the Noëlaerhabdaceae rooted with both *T. lutea* based on an alignment of 2930 concatenated orthologous proteins (1,915,024 residues, of which 821,172 residues were retained after ambiguous region removal). All trees are drawn to scale, with branch lengths measured in number of substitutions per site (scale bar) and nodes with bootstraps values of 100%.

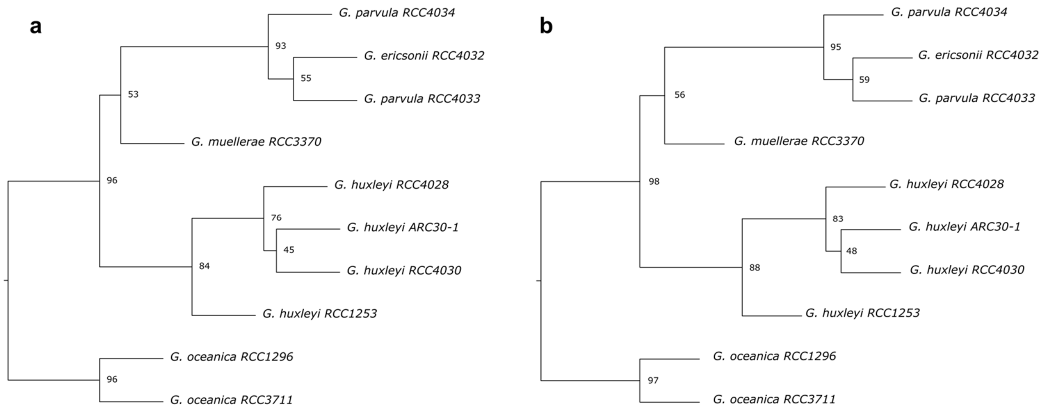

Supplementary Figure 4 | Species trees of the Noëlaerhabdaceae inferred with a multi coalescent approach. The analyses are based on sequenced *Gephyrocapsa* strains, using 6,278 phylogenies constructed from genomic fragments 5kb (**a**) and 10kb long (**b**). Branch lengths are meaningless – only the tree topology is informative. Values next to nodes refer to quartet support as the percentage of quartets in gene trees supporting a node.

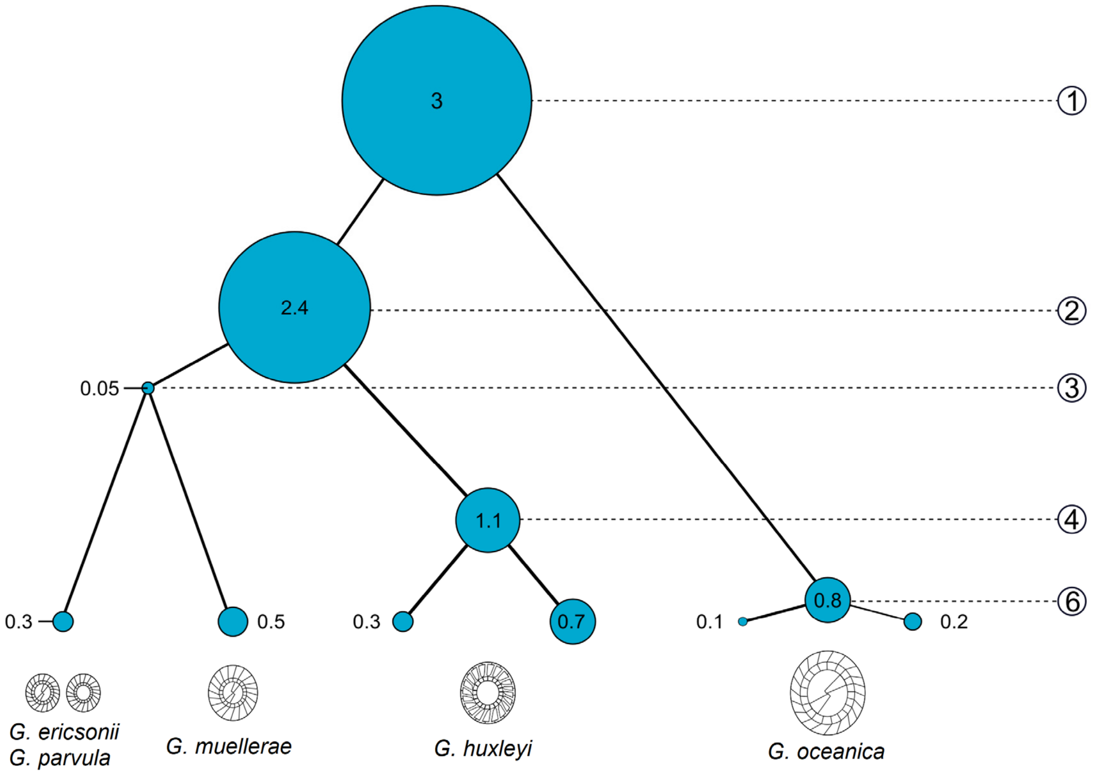

Supplementary Figure 5 | Demographic modelling of *Gephyrocapsa* speciation history without migration bands (i.e. no migration allowed). The sizes of blue circles are proportional to estimated population sizes of extant and ancestral *Gephyrocapsa* species; the numbers inside blue circles (=θ/θ_{Ghux}) show population sizes relative to that in extant *G. huxleyi*.

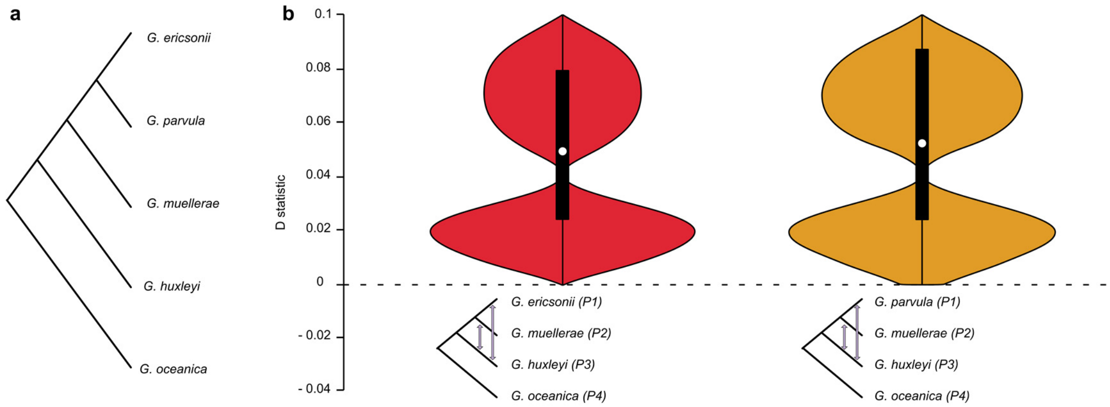

Supplementary Figure 6 | ABBA-BABA tests for introgression. (**a**) Phylogeny of morphospecies used for ABBA-BABA tests. (**b**) Violin plots show the distributions of Patterson's *D*-statistics from strain-based permutations using different taxa combinations, as labeled in the phylogeny.

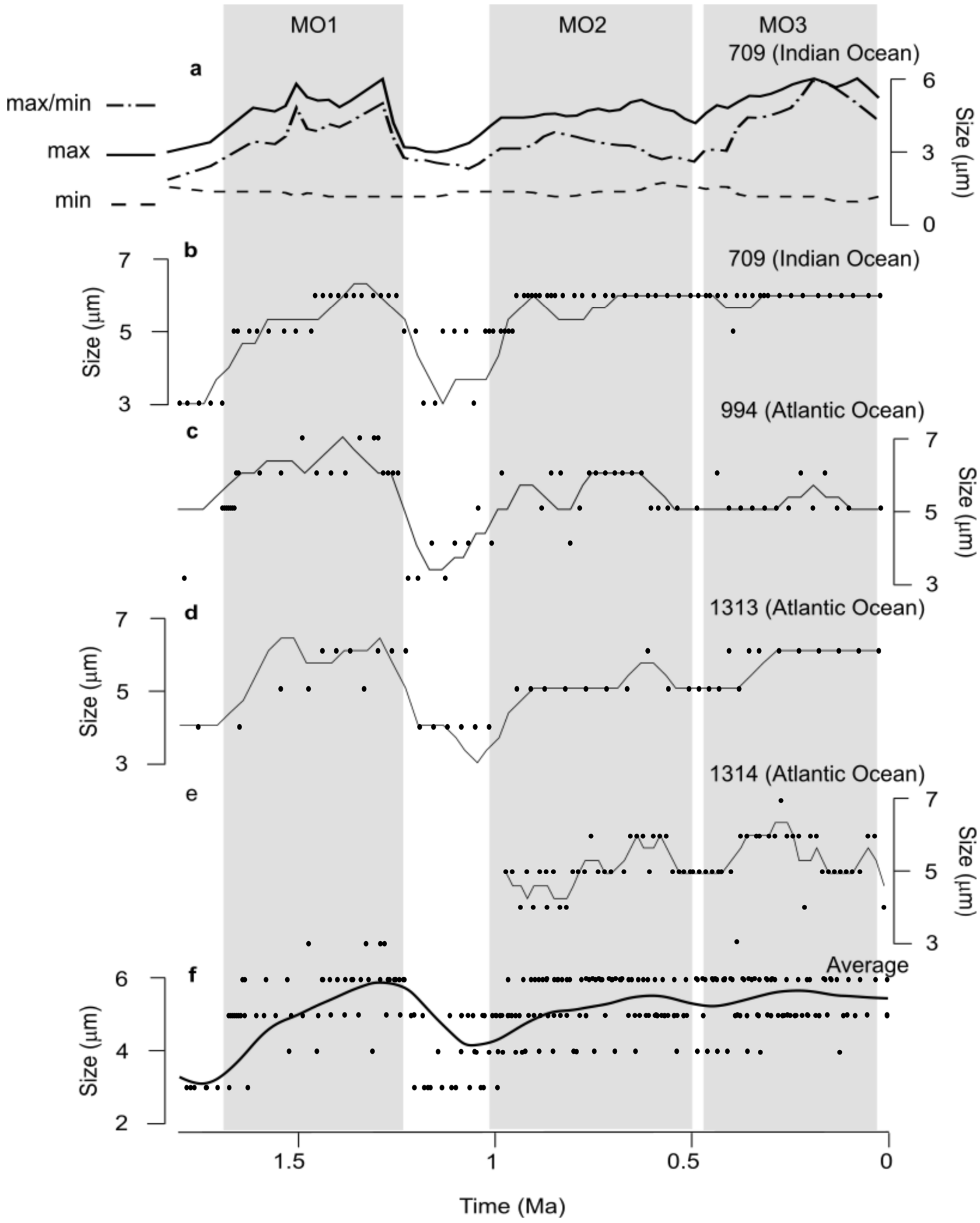

Supplementary Figure 7 | MO cycles in different sediment cores over 1.8 Ma. (**a**)-(**b**) Size variation of coccoliths in *Gephyrocapsa* spp. at site 709. a, Coccolith size variations. b, Variation of coccolith size range. (**c**) Variation of coccolith size range at site 994. (**d**) Variation of coccolith size range at site 1313. (**e**) Variation of coccolith size range at site 1314. (**f**) Average variation of coccolith size range. All curves are smoothed using 3 point-average.

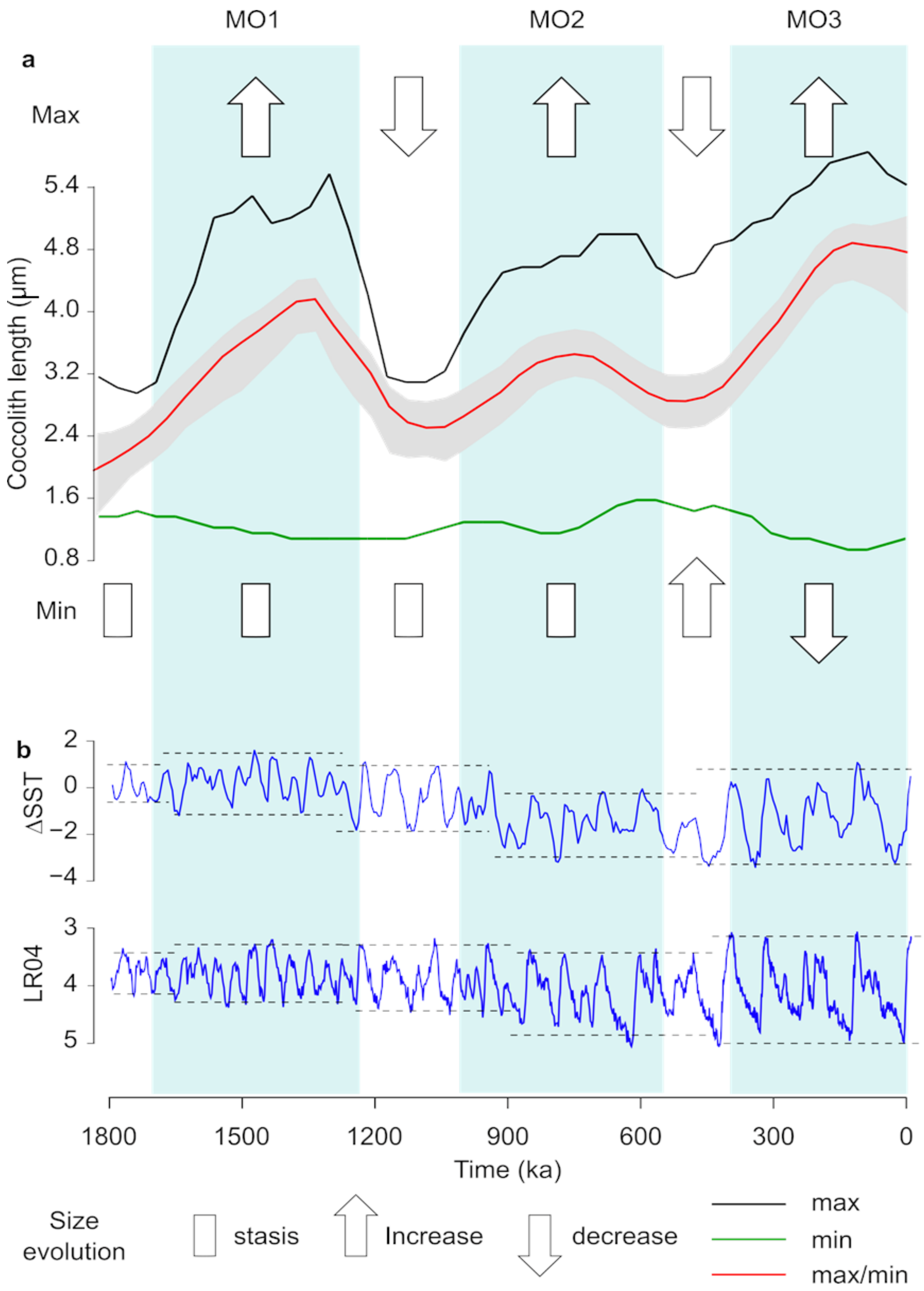

Supplementary Figure 8 | Characterisation of coccolith size evolution and definition of the MO cycles over 1.8 Ma at site 709. (**a**) Size variation of coccoliths in *Gephyrocapsa* spp. at site 709. Max/min corresponds to size variance. (**b**) Δ sea surface temperature (ΔSST)[2] and LR04[3] over 1800 ka.

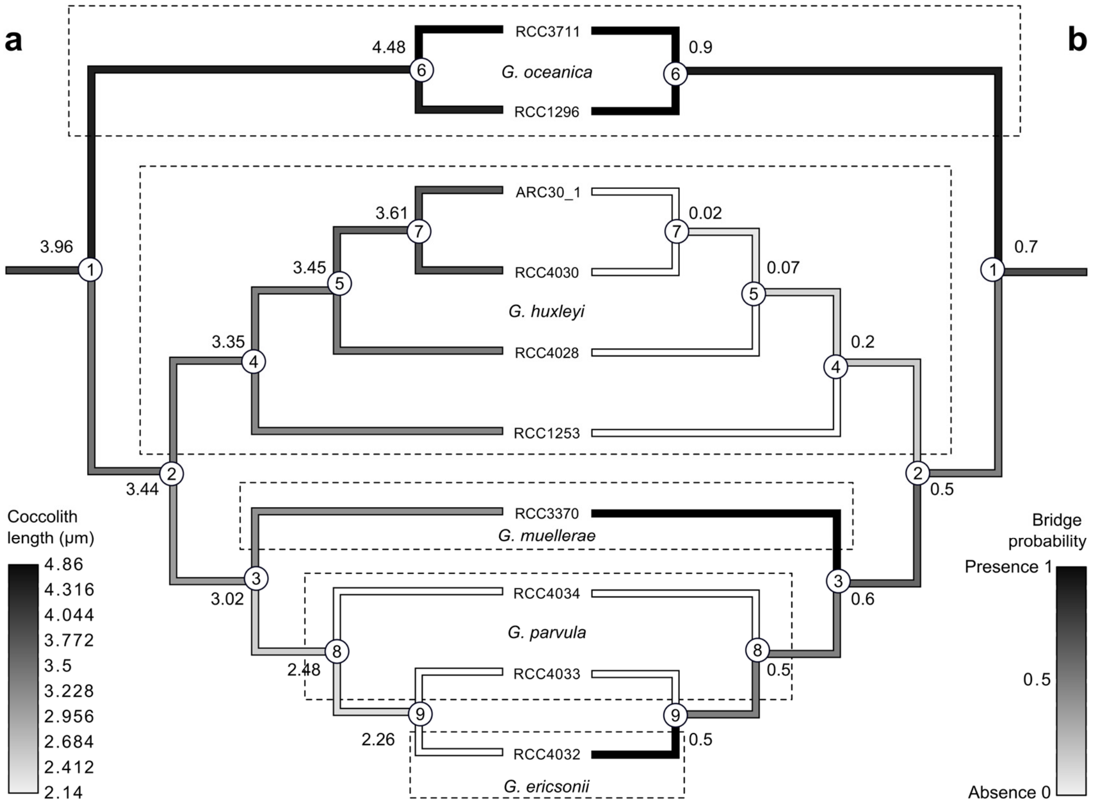

Supplementary Figure 9 | Ancestral reconstruction for traits of coccolith length (a) and bridge loss and gain (b). The model of reconstruction was parsimony as implemented in Mesquite (v.3.02)[4], and ancestral state reconstruction was estimated using the inferred species tree topology. For coccolith length, ancestral reconstruction for length measure is given next to each node in µm. For bridge presence, ancestral probability given next to each node. The numbers in circles at each node show node numbers as in figure 2a. Morphospecies are delimited with dashed squares.

Supplementary Tables

Supplementary Table 1. List of strains used in this study.

RCC code	Other code	Accession	Species	Region of Isolation	Latitude	Longitude	Raw data, Gb	% reads mapped
	ARC30.1	SRR8885247	*G. huxleyi*	N.E. Atlantic	79.00	8.00	12.21	69%
RCC4028	CHC350	ERR695589	*G. huxleyi*	S.W. Pacific	-30.25	-71.70	17.04	63%
RCC4030	CHC428	ERR695590	*G. huxleyi*	S.W. Pacific	-16.75	-86.00	13.10	62%
RCC1253	OS2	SRR8885248	*G. huxleyi*	N.E. Pacific	43.22	141.02	19.31	56%
RCC1296	ESP56	SRR8885245	*G. oceanica*	Mediterranean	36.41	-4.25	19.02	44%
RCC3370	CHC184	SRR8885246	*G. muellerae*	S.W. Pacific	-30.25	-71.70	14.95	45%
RCC3711	TS3	SRR8885243	*G. oceanica*	N.E. Pacific	33.15	133.38	12.59	40%
RCC4032	CHC516	SRR8885244	*G. ericsonii*	S.W. Pacific	-20.77	-70.66	12.51	42%
RCC4033	CHC527	SRR8885241	*G. parvula*	S.W. Pacific	-20.77	-70.66	7.79	41%
RCC4034	CHC528	SRR8885242	*G. parvula*	S.W. Pacific	-20.77	-70.66	12.67	40%

Supplementary Table 2. Estimates of population-scaled mutation rate (θ) for extant species and ancestral nodes.

**Sample**	**θ_Geric**	**θ_Gmuel**	**θ_Ghux**	**θ_Goce**	**θ_node3**	**θ_node2**	**θ_node1**
mean	12.5596	23.4706	78.6926	34.019	3.9153	97.5932	118.0754
std err. of mean	0.0311	0.0228	0.0418	0.0102	0.1872	0.2094	0.2056
std. dev	0.6414	1.1144	1.9731	0.9749	2.8974	5.5143	5.9698
variance	0.4114	1.242	3.893	0.9504	8.3949	30.408	35.638
95% HPD Interval	[11.2902, 13.7694]	[21.2903, 25.6516]	[74.78, 82.4987]	[32.1155, 35.9472]	[0.129, 9.9052]	[86.9959, 108.6092]	[106.3266, 129.7574]
Effective sample size (ESS)	424.979	2379.745	2225.743	9047.846	239.58	693.4739	843.4315

Supplementary Table 3. Inferred “migration bands” in the G-PhoCS analysis of gene flow.

Sample	m_node2->Goce	m_Goce->node2	m_node3->Goce	m_Goce->node3	m_Gmuel->Goce	m_Goce->Gmuel	m_Geric->Goce	m_Goce->Geric	m_node3->Ghux
mean	7.0869	9.20E-03	0.0476	0.7016	0.115	6.49E-03	0.2132	2.22E-03	14.2036
std error of mean	0.0767	8.77E-04	7.15E-03	0.1311	3.33E-03	1.04E-03	3.01E-03	3.08E-04	2.1373
std dev.	1.6148	0.0137	0.1107	2.0303	0.0613	0.016	0.0705	4.68E-03	32.4103
variance	2.6075	1.87E-04	0.0123	4.1222	3.76E-03	2.55E-04	4.96E-03	2.19E-05	1050.425
95% HPD Interval	[3.8606, 10.2352]	[0, 0.0382]	[1E-5, 0.2387]	[0, 3.4537]	[3.77E-3, 0.2252]	[0, 0.0404]	[0.0809, 0.3481]	[0, 0.0122]	[3.87E-3, 71.1619]
effective sample size (ESS)	443.4269	242.9071	239.5444	239.9317	338.2861	236.8427	547.3385	231.6849	229.9488

Supplementary Table 3 (continued)

Sample	m_Ghux->node3	m_Ghux->Goce	m_Goce->Ghux	m_Gmuel->Ghux	m_Ghux->Gmuel	m_Geric->Ghux	m_Ghux->Geric	m_Geric->Gmuel	m_Gmuel->Geric
mean	1.0456	0.5207	9.24E-04	6.06E-03	1.1614	0.0602	0.2844	9.68E-03	0.8352
std error of mean	0.3458	4.07E-03	1.75E-04	1.04E-03	7.03E-03	3.64E-03	0.023	1.65E-03	9.57E-03
std dev.	5.1574	0.1156	2.65E-03	0.0156	0.2136	0.0564	0.337	0.0252	0.2112
variance	26.5991	0.0134	7.01E-06	2.43E-04	0.0456	3.18E-03	0.1136	6.36E-04	0.0446
95% HPD Interval	[0, 2.4485]	[0.3018, 0.7497]	[0, 6.44E-3]	[0, 0.0424]	[0.7611, 1.5859]	[0, 0.1562]	[0, 0.8595]	[0, 0.0572]	[0.4266, 1.2355]
effective sample size (ESS)	222.4326	805.5747	227.6554	222.6145	923.2261	240.3979	214.2736	234.9397	487.2653

Supplementary Table 4. ABBA/BABA tests for interspecific gene flow.

H1	H2	H3	Out	nABBA	nBABA	D	SD	Z	SD
G. ericsonii (RCC4032)	*G. muellereae (RCC5119)*	*E. huxleyi (RCC1253)*	*G. oceanica (RCC1296)*	17813	14898	**0.089**	0.006	16.117	0.010
G. ericsonii (RCC4032)	*G. muellereae (RCC5119)*	*E. huxleyi (ARC30_1)*	*G. oceanica (RCC1296)*	16496	14539	**0.063**	0.006	11.109	0.009
G. ericsonii (RCC4032)	*G. muellereae (RCC5119)*	*E. huxleyi (RCC4028)*	*G. oceanica (RCC1296)*	15513	14797	**0.024**	0.006	4.113	0.009
G. ericsonii (RCC4032)	*G. muellereae (RCC5119)*	*E. huxleyi (RCC4030)*	*G. oceanica (RCC1296)*	15065	14329	**0.025**	0.006	4.293	0.009
G. parvula (RCC4034)	*G. muellereae (RCC5119)*	*E. huxleyi (RCC1253)*	*G. oceanica (RCC1296)*	14685	12165	**0.094**	0.006	15.379	0.008
G. parvula (RCC4034)	*G. muellereae (RCC5119)*	*E. huxleyi (ARC30_1)*	*G. oceanica (RCC1296)*	13902	12235	**0.064**	0.006	10.311	0.008
G. parvula (RCC4034)	*G. muellereae (RCC5119)*	*E. huxleyi (RCC4028)*	*G. oceanica (RCC1296)*	12789	12259	**0.021**	0.006	3.349	0.008
G. parvula (RCC4034)	*G. muellereae (RCC5119)*	*E. huxleyi (RCC4030)*	*G. oceanica (RCC1296)*	12524	11984	**0.022**	0.006	3.449	0.008
G. parvula (RCC4033)	*G. muellereae (RCC5119)*	*E. huxleyi (RCC1253)*	*G. oceanica (RCC1296)*	15681	13032	**0.092**	0.006	15.633	0.008
G. parvula (RCC4033)	*G. muellereae (RCC5119)*	*E. huxleyi (ARC30_1)*	*G. oceanica (RCC1296)*	14887	13037	**0.066**	0.006	11.071	0.008
G. parvula (RCC4033)	*G. muellereae (RCC5119)*	*E. huxleyi (RCC4028)*	*G. oceanica (RCC1296)*	13705	13073	**0.024**	0.006	3.862	0.008
G. parvula (RCC4033)	*G. muellereae (RCC5119)*	*E. huxleyi (RCC4030)*	*G. oceanica (RCC1296)*	13416	12690	**0.028**	0.006	4.493	0.008
G. ericsonii (RCC4032)	*G. muellereae (RCC5119)*	*E. huxleyi (RCC1253)*	*G. oceanica (RCC3711)*	17278	14562	**0.085**	0.006	15.221	0.009
G. ericsonii (RCC4032)	*G. muellereae (RCC5119)*	*E. huxleyi (ARC30_1)*	*G. oceanica (RCC3711)*	15935	14065	**0.062**	0.006	10.796	0.009
G. ericsonii (RCC4032)	*G. muellereae (RCC5119)*	*E. huxleyi (RCC4028)*	*G. oceanica (RCC3711)*	15218	14418	**0.027**	0.006	4.647	0.009
G. ericsonii (RCC4032)	*G. muellereae (RCC5119)*	*E. huxleyi (RCC4030)*	*G. oceanica (RCC3711)*	14642	13947	**0.024**	0.006	4.110	0.009
G. parvula (RCC4034)	*G. muellereae (RCC5119)*	*E. huxleyi (RCC1253)*	*G. oceanica (RCC3711)*	14320	11924	**0.091**	0.006	14.790	0.008
G. parvula (RCC4034)	*G. muellereae (RCC5119)*	*E. huxleyi (ARC30_1)*	*G. oceanica (RCC3711)*	13433	11837	**0.063**	0.006	10.040	0.008
G. parvula (RCC4034)	*G. muellereae (RCC5119)*	*E. huxleyi (RCC4028)*	*G. oceanica (RCC3711)*	12551	11922	**0.026**	0.006	4.021	0.008
G. parvula (RCC4034)	*G. muellereae (RCC5119)*	*E. huxleyi (RCC4030)*	*G. oceanica (RCC3711)*	12113	11677	0.018	0.006	2.827	0.007
G. parvula (RCC4033)	*G. muellereae (RCC5119)*	*E. huxleyi (RCC1253)*	*G. oceanica (RCC3711)*	15213	12775	**0.087**	0.006	14.573	0.008
G. parvula (RCC4033)	*G. muellereae (RCC5119)*	*E. huxleyi (ARC30_1)*	*G. oceanica (RCC3711)*	14327	12637	**0.063**	0.006	10.292	0.008
G. parvula (RCC4033)	*G. muellereae (RCC5119)*	*E. huxleyi (RCC4028)*	*G. oceanica (RCC3711)*	13427	12755	**0.026**	0.006	4.153	0.008

Supplementary Table 5. Morphometric features of strains used in this study.

Species	Morphotype	RCC code	Other strain code	Coccolith length (um)	SD	Bridge angle	SD
G.oceanica	GL	RCC1296	ESP56	4.6	0.49	58.2°	9.11
G.oceanica	GL	RCC3711	TS3	4.86	0.52	64.21°	8.23
G.huxleyi	A		ARC30-1	3.61	0.43		
G.huxleyi	A	RCC4028	CHC350	3.79	0.42		
G.huxleyi	A	RCC4030	CHC428	3.39	0.41		
G.huxleyi	O	RCC1253	OS2	3.16	0.31		
G.muellerae	GO	RCC3370	CHC184	3.29	0.38	31.89°	7.09
G.ericsonii	GM	RCC4032	CHC516	2.16	0.29	42.12°	7.36
G.parvula	parvula	RCC4033	CHC527	2.14	0.25		
G.parvula	parvula	RCC4034	CHC528	2.16	0.29		

Supplementary Table 6. Relaxed molecular clock-based estimates for species divergence time in genus *Gephyrocapsa*.

Relaxed clock	rgene_gamma		node 1 age (Ma)		node 2 age (Ma)		node 3 age (Ma)	
	α	β	mean	95% HPD CI	mean	95% HPD CI	mean	95% HPD CI
uncorrelated	1	10	0.549	0.493 - 0.604	0.322	0.289 - 0.351	0.285	0.257 - 0.311
uncorrelated	1	100	0.556	0.496 - 0.605	0.325	0.292 - 0.352	0.289	0.258 - 0.312
uncorrelated	1	500	0.540	0.487 - 0.603	0.316	0.287 - 0.350	0.280	0.255 - 0.311
autocorrelated	1	10	0.537	0.472 - 0.587	0.325	0.289 - 0.352	0.289	0.257 - 0.313
autocorrelated	1	100	0.566	0.531 - 0.594	0.342	0.322 - 0.355	0.304	0.287 - 0.317
autocorrelated	1	500	0.567	0.515 - 0.597	0.342	0.313 - 0.356	0.305	0.279 - 0.317

Supplementary Table 7. Morphometric features of main morphospecies from MO cycles

MO event	Species	group	Coccolith length (um)	Bridge angle	Central opening*	Remarks
**1**	***Gephyrocapsa lumina* Burky (.sp B Matsuoka and Okada)**	**large**	**5 - 7**	**40-60°**	**small**	sometimes referred to as large *Gephyrocapsa* and *G. oceanica***
**2**	***Gephyrocapsa omega* Burky (.sp C Matsuoka and Okada)**	**large**	**4 - 7**	**65-90°**	**large**	sometimes referred to as large *Gephyrocapsa* and *G. oceanica***
	Gephyrocapsa aperta Kamptner 1963	small	1.7 - 2.3	23-32°	52-60	sometimes referred to as small *Gephyrocapsa***
**3**	***Gephyrocapsa oceanica* Kamptner (.sp D Matsuoka and Okada)**	**large**	**3.5 - 6**	**>40°**	**44 - 50**	includes *G. margereli*, sometimes referred to as large *Gephyrocapsa***
	Gephyrocapsa huxleyi Lohmann	*G. huxleyi*	2 - 5	na	variable	
	Gephyrocapsa muellerae Breheret	medium	2.6 - 4.1	5-37°	44 - 47	includes *G. margereli*, included medium *Gephyrocapsa*
	Gephyrocapsa ornata Heimdal	small	2.2 - 3	10-20°	?	included in small *Gephyrocapsa*
	Gephyrocapsa ericsonii McIntyre and Be	small	1.4 -2.3	15-55°	44 - 47	included in small *Gephyrocapsa*
	Gephyrocapsa parvula McIntyre and Be	small	~2	na	variable	

*Central opening = (length of central area/coccolith length) x 100

**Species names in the fossil record are not always consistent across the studies.

Supplementary References

1 Bendif, E. M. *et al.* Recent reticulate evolution in the ecologically dominant lineage of coccolithophores. *Frontiers in Microbiology* **7**, 784 (2016).

2 Martínez-Botí, M. A. *et al.* Plio-Pleistocene climate sensitivity evaluated using high-resolution CO2 records. *Nature* **518**, 49-54 (2015).

3 Lisiecki, L. E. & Raymo, M. E. A Pliocene-Pleistocene stack of 57 globally distributed benthic δ18O records. *Paleoceanography* **20**, 1-17 (2005).

4 The Mesquite Project Team, *Documentation for Mesquite: a modular system for evolutionary analysis.*, < http://www.mesquiteproject.org> (2018).
