# Supplementary material for: Repeated species radiations in the recent evolution of the key marine phytoplankton lineage Gephyrocapsa
Source: Nat Commun. 2019 Sep 17;10:4234. doi: 10.1038/s41467-019-12169-7 (PMC6748936; doi:10.1038/s41467-019-12169-7)
Supplement: Supplementary file 3 — Reporting Summary [file 41467_2019_12169_MOESM3_ESM.pdf]

natureresearch

Corresponding author(s): Dmitry A. Filatov

Last updated by author(s): Aug 17, 2019

# Reporting Summary

Nature Research wishes to improve the reproducibility of the work that we publish. This form provides structure for consistency and transparency in reporting. For further information on Nature Research policies, see Authors & Referees and the Editorial Policy Checklist .

## Statistics

For all statistical analyses, confirm that the following items are present in the figure legend, table legend, main text, or Methods section.

| n/a | Confirmed | |
|---|---|---|
| ☒ | ☐ | The exact sample size (*n*) for each experimental group/condition, given as a discrete number and unit of measurement |
| ☒ | ☐ | A statement on whether measurements were taken from distinct samples or whether the same sample was measured repeatedly |
| ☐ | ☒ | The statistical test(s) used AND whether they are one- or two-sided<br>*Only common tests should be described solely by name; describe more complex techniques in the Methods section.* |
| ☒ | ☐ | A description of all covariates tested |
| ☐ | ☒ | A description of any assumptions or corrections, such as tests of normality and adjustment for multiple comparisons |
| ☐ | ☒ | A full description of the statistical parameters including central tendency (e.g. means) or other basic estimates (e.g. regression coefficient) AND variation (e.g. standard deviation) or associated estimates of uncertainty (e.g. confidence intervals) |
| ☐ | ☒ | For null hypothesis testing, the test statistic (e.g. *F*, *t*, *r*) with confidence intervals, effect sizes, degrees of freedom and *P* value noted<br>*Give P values as exact values whenever suitable.* |
| ☐ | ☒ | For Bayesian analysis, information on the choice of priors and Markov chain Monte Carlo settings |
| ☒ | ☐ | For hierarchical and complex designs, identification of the appropriate level for tests and full reporting of outcomes |
| ☒ | ☐ | Estimates of effect sizes (e.g. Cohen's *d*, Pearson's *r*), indicating how they were calculated |

*Our web collection on statistics for biologists contains articles on many of the points above.*

## Software and code

Policy information about availability of computer code

| | |
|---|---|
| Data collection | Morphometric measurements were carried out using ImageJ software (http://imagej.nih.gov/ij/) |
| Data analysis | For fig1a data of monthly sea surface temperatures (2002–2017) were obtained from the Modis Aqua (https://oceancolor.gsfc.nasa.gov/) and plotted using SeaDAS (https://seadas.gsfc.nasa.gov/).<br><br>Raw sequence reads were trimmed with Trimmomatic version 0.33 (program described in reference 70) available from http://www.usadellab.org/cms/?page=trimmomatic<br>Read mapping to reference was done with bwa-mem version 0.7.12-r1039 (program described in reference 72) available from http://bio-bwa.sourceforge.net/)<br>Duplicated reads were removed using Picard version 2 (https://broadinstitute.github.io/picard/)<br>To process the mapped reads and call SNPs, as described in the methods, we used Genome Analyses Toolkit version 3.4-46 (program described in reference 73) available from https://software.broadinstitute.org/gatk/<br>SNPs were converted into fasta multiple alignment sequences using a custom script vcf2fas available at https://github.com/brunonevado/vcf2fas<br>For phylogenetic maximum likelihood inference we used RAxML version 8 (program described in reference 74) available from https://cme.h-its.org/exelixis/web/software/raxml/<br>For species phylogeny reconstruction from gene trees we used ASTRAL version 5.6.3 (program described in reference 75) available from https://github.com/smirarab/ASTRAL<br>Orthologs were identified using Orthofinder version 2.2.6 (program described in reference 78) available from http://www.stevekellylab.com/software/orthofinder<br>Alignments with outgroup sequences were done using MAFFT version 7 (program described in reference 79) available at https://mafft.cbrc.jp/alignment/software/<br>Molecular clock-based analyses were conducted with mcmctree program from the PAML package version 4.9b (program described in |

reference 80. Available from http://abacus.gene.ucl.ac.uk/software/paml.html)
The convergence of MCMC runs was monitored using Tracer version 1.5 (program described in reference 82) available at https://beast.community/tracer
DensiTree plot was produced using Densitree version 2.2.1 (program described in reference 83; available from https://www.cs.auckland.ac.nz/~remco/DensiTree/).
Shimodaira–Hasegawa tests were conducted in CONSEL version 0.2 (program available from http://stat.sys.i.kyoto-u.ac.jp/prog/consel/)
Phylogenies for Consel analysis were inferred with PhyML version 3.086 (available at http://www.atgc-montpellier.fr/phyml/)
The analysis of demographic history was conducted with program G-PhoCS version 1.2.3 (program described in reference 40; available from https://omictools.com/g-phocs-tool )
Patterson's D-statistic test was conducted with a custom script available at https://github.com/brunonevado/calcD_from_fas

For manuscripts utilizing custom algorithms or software that are central to the research but not yet described in published literature, software must be made available to editors/reviewers. We strongly encourage code deposition in a community repository (e.g. GitHub). See the Nature Research guidelines for submitting code & software for further information.

## Data

Policy information about availability of data

All manuscripts must include a data availability statement. This statement should provide the following information, where applicable:

- Accession codes, unique identifiers, or web links for publicly available datasets
- A list of figures that have associated raw data
- A description of any restrictions on data availability

Data availability: All genome sequencing data generated in this study have been deposited in the National Center for Biotechnology Information (NCBI; https://www.ncbi.nlm.nih.gov) and are accessible under bioproject number PRJNA532411. Two previously published G. huxleyi genomes (RCC4028 and RCC4030) that were used in this study are available from NCBI under accession numbers ERR695589 and ERR695590. Coccoliths morphometric data analysed in this study is available from Pangaea database (https://doi.pangaea.de/10.1594/PANGAEA.903745).

# Field-specific reporting

Please select the one below that is the best fit for your research. If you are not sure, read the appropriate sections before making your selection.

☐ Life sciences ☐ Behavioural & social sciences ☒ Ecological, evolutionary & environmental sciences

For a reference copy of the document with all sections, see nature.com/documents/nr-reporting-summary-flat.pdf

# Ecological, evolutionary & environmental sciences study design

All studies must disclose on these points even when the disclosure is negative.

| | |
|---|---|
| Study description | The study sequenced the genomes of 8 strains of microplankton Gephyrocapsa + used 2 published genomes to reconstruct the phylogenetic relationships in the genus and link it to extensive fossil record available for this genus. |
| Research sample | The study is based on the analysis of genomes from 10 strains of microplankton strains from genus Gephyrocapsa (listed in table S1). All strains were obtained from Roscoff culture collection, http://roscoff-culture-collection.org/ Also, we used morphometric data from a previous study (reference 30); this data is available from Pangaea database, https://www.pangaea.de/ |
| Sampling strategy | As this is a phylogenetic study, we aimed to analyse at least 1 sample per each of the Gephyrocapsa species. |
| Data collection | The cultures of the strains were obtained from Roscoff culture collection and DNA was extracted as described in the methods. Genomic DNA was sequenced using Illumina at the WTCHG Oxford genomic facility. |
| Timing and spatial scale | The samples analysed come from all over the world oceans (see Figure 1A in the manuscript); these samples were isolated over the last ~10 years by Roscoff culture collection. |
| Data exclusions | no data was excluded from analysis |
| Reproducibility | To test reproducibility the analyses were repeated several times. In particular, the MCMC-based timing of the species splits in the phylogeny were repeated multiple times with different parameter values, as detailed in table S6. |

| | |
|---|---|
| Randomization | This is not directly relevant to this study - we analysed only 10 Gephyrocapsa strains and the only "grouping" we've done was phylogenetic tree reconstruction, as described in the paper. |
| Blinding | Blinding is not relevant for this study. This is a phylogenetic study and blinding is not applicable in this context. |

Did the study involve field work? ☐ Yes ☒ No

# Reporting for specific materials, systems and methods

We require information from authors about some types of materials, experimental systems and methods used in many studies. Here, indicate whether each material, system or method listed is relevant to your study. If you are not sure if a list item applies to your research, read the appropriate section before selecting a response.

## Materials & experimental systems

| n/a | Involved in the study | |
|---|---|---|
| ☒ | ☐ | Antibodies |
| ☒ | ☐ | Eukaryotic cell lines |
| ☐ | ☒ | Palaeontology |
| ☒ | ☐ | Animals and other organisms |
| ☒ | ☐ | Human research participants |
| ☒ | ☐ | Clinical data |

## Methods

| n/a | Involved in the study | |
|---|---|---|
| ☒ | ☐ | ChIP-seq |
| ☒ | ☐ | Flow cytometry |
| ☒ | ☐ | MRI-based neuroimaging |

# Palaeontology

| | |
|---|---|
| Specimen provenance | All our palaeonthological data come from previously published work (reference 30), thus no permits were necessary. The locations of the ocean floor drilling cores used by previous study and re-analysed in our work are shown with stars on figure 1 in the manuscript. |
| Specimen deposition | No new palaentological samples were used in this study, so no specimens were deposited. |
| Dating methods | As we used published data, the dating was done in the previous studies (reference 30). |

☒ Tick this box to confirm that the raw and calibrated dates are available in the paper or in Supplementary Information.
